# Identifying circRNA-associated-ceRNA networks in juvenile spondyloarthropathies patients

**DOI:** 10.1186/s12969-023-00855-2

**Published:** 2023-07-28

**Authors:** Wei Qijiao, Zhang Tao, Liu Haimei, Li Guomin, Sun Li

**Affiliations:** Department of Rheumatology, Children’s Hospital of Fudan University, Shanghai, China

**Keywords:** Juvenile spondyloarthropathies, circRNA, miRNA, mRNA, Competing endogenous RNAs network, Bioinformatics analysis

## Abstract

**Background:**

Juvenile spondyloarthropathies (JSpA) are defined as a heterogeneous group of diseases that start before the age of 16. The study aimed to identify key genes and pathways that are influenced by circRNAs and to screen potential therapeutic agents for JSpA. The study involved the analysis of circRNA expression profiles, identification of circRNA-miRNA-mRNA regulatory networks, and functional annotation of differentially expressed genes. The results of the study may have provided insights into the molecular mechanisms underlying JSpA and potential therapeutic targets for this disease.

**Methods:**

In this study, sequencing data of circRNA, miRNA, and mRNA were obtained from the GEO datasets. The data were then analyzed to identify candidates for constructing a circRNA-miRNA-mRNA network based on circRNA-miRNA interactions and miRNA-mRNA interactions. Functional enrichments of genes were performed using the DAVID database. A PPI network was constructed using the STRING database and visualized using Cytoscape software. The MCODE plugin app was used to explore hub genes in the PPI network. The expression changes in immune cells were assessed using the online CIBERSORT algorithm to obtain the proportion of various types of immune cells. Finally, the Connectivity Map L1000 platform was used to identify potential agents for JSpA treatment. Overall, this study aimed to provide a comprehensive understanding of the molecular mechanisms underlying JSpA and to identify potential therapeutic agents for this disease.

**Results:**

A total of 225 differentially expressed circRNAs (DEcircRNAs), 23 differentially expressed miRNAs (DEmiRNAs) and 1324 differentially expressed mRNAs (DEmRNAs) were identified. We integrated 5 overlapped circRNAs, 7 miRNAs and 299 target mRNAs into a circRNA–miRNA–mRNA network. We next identified 10 hub genes based on the PPI network. KEGG pathway analysis revealed that the DEGs were mainly associated with JAK-STAT signal pathway. We found that neutrophils accounted for the majority of all enriched cells. In addition, we discovered several chemicals as potential treatment options for JSpA.

**Conclusions:**

Through this bioinformatics analysis, we suggest a regulatory role for circRNAs in the pathogenesis and treatment of JSpA from the view of a competitive endogenous RNA (ceRNA) network.

**Supplementary Information:**

The online version contains supplementary material available at 10.1186/s12969-023-00855-2.

## Background

Juvenile spondyloarthropathies (JSpA) are a complex and heterogeneous group of diseases that typically manifest before the age of 16. JSpA is characterized by peripheral joint involvement, axial skeletal involvement, enthesitis, and HLA-B27 positivity. The different subtypes of JSpA include juvenile ankylosing spondylitis, psoriatic arthritis, reactive arthritis, inflammatory bowel disease-associated arthritis, seronegative enthesopathy arthropathy syndrome, and enthesitis-associated arthritis [1]. Despite significant efforts to understand the pathogenesis and molecular mechanisms of JSpA, they are not yet fully understood [2]. Therefore, further research is necessary to explore the molecular characteristics and underlying mechanisms of the disease.

Circular RNA (circRNA) is a type of endogenous noncoding RNA that was only recently discovered. These molecules have a covalently closed loop structure and are formed by back splicing. CircRNAs are highly abundant, stable, and exhibit tissue/stage specificity, as well as species conservation [3]. Recent evidence has suggested that circRNAs have a crucial role in rheumatic diseases, including spondyloarthritis. CircRNAs have been studied as potential diagnostic markers and therapeutic molecules in these diseases. In particular, ceRNA regulation has been found to play an important role in ankylosing spondylitis (AS), which is a subtype of JSpA [4]. Several studies have investigated the regulatory role of circRNAs in AS, highlighting the potential of these molecules as therapeutic targets for the disease [5–9].

Integrative ceRNA regulatory networks, which incorporate data on circRNA, miRNA, and mRNA, can provide valuable insights into complex gene interactions and help identify potential biomarkers for the diagnosis and treatment of diseases such as JSpA. The Gene Expression Omnibus (GEO) database provides a rich source of RNA sequencing data on circRNA, miRNA, and mRNA, making it an excellent resource for data mining and biological discovery. Constructing integrative ceRNA regulatory networks based on GEO database can lead to the identification of more accurate prognostic markers for JSpA and other diseases. However, few studies have explored the use of GEO database to construct integrative ceRNA regulatory networks, highlighting the need for further research in this area.

In this current research, the GEO database was utilized to identify DEcircRNAs, DEmiRNAs, and DEmRNAs in peripheral blood mononuclear cells (PBMCs) of JSpA patients. Interactions between miRNA-circRNA and miRNA-mRNA were determined using website tools, and a ceRNA network was constructed. Functional enrichment analyses were conducted to assess the biological functions of DEmRNAs, and hub genes were identified through a PPI network. The expression changes in immune cells were assessed using the online CIBERSORTx algorithm, and the proportion of various types of immune cells was determined. Finally, a connectivity map (CMap) analysis was performed to identify potential compounds that could be useful in the treatment of JSpA. The use of GEO databases in this research helped ensure the accuracy and repeatability of the analysis results.

## Methods

### Data collection

The NCBI GEO is a public functional genomics data repository that contains high-throughput gene expression data, chips, and microarrays [10]. We conducted a search using the keywords “juvenile idiopathic arthritis” in the GEO Database, resulting in 805 records. After narrowing down the search to include only “Homo sapiens,“ the number of records reduced to 801. The objective of this study is to investigate circRNA-associated-ceRNA networks in patients with juvenile spondyloarthropathies. To obtain the mRNA dataset, we specifically selected the study type “Expression profiling by array,“ which yielded 35 remaining records. We carefully examined these 35 links and identified GSE58667 (11 JSpA patients and 4 controls) [11] as the gene expression dataset for juvenile spondyloarthritis, making it our selection. For the miRNA dataset, we utilized the study type “Non-coding RNA profiling by array,“ resulting in four remaining records. Among these, GSE79481 (8 enthesitis-related arthritis patients and 8 controls) [12] was chosen as it provided global miRNA profiling in patients with enthesitis-related arthritis. Regarding the circRNA dataset, we performed a search using the terms “spondylitis” and “circRNA,“ along with the filters “Homo sapiens” and “Non-coding RNA profiling by array,“ resulting in two remaining links. GSE178408 (6 ankylosing spondylitis patients and 3 controls) [7] was selected as it contained circRNA expression profiles from ankylosing spondylitis patients and healthy controls. The children included in the GSE58667 dataset were diagnosed with JSpA based on the criteria set forth by the International League of Associations for Rheumatology (ILAR). The patients with ERA in the GSE79481 dataset met the ILAR criteria for diagnosis. Lastly, the patients in the GSE178408 dataset were diagnosed with ankylosing spondylitis (AS) according to the modified New York criteria established in 1984.

### Identification of DEcircRNAs, DEmiRNAs and DEmRNAs

We utilized the GEO2R web-based tool [13] to detect DEGs in the samples from diseased individuals and control subjects. The DEcircRNAs were identified based on the criteria of |log2(fold-change)| > 2.0 and P-value < 0.05. Similarly, significant DEmiRNAs and DEmRNAs were recognized using the standards of |log2(fold-change)| > 1.0 and P-value < 0.05.

### Prediction of miRNA and mRNA

To predict the target miRNAs of DEcircRNAs, we referred to the Circbank database [14]. The target mRNAs of miRNAs were retrieved from the miRDB databases [15]. To ensure the credibility of the findings, we identified the final miRNAs by selecting overlapping miRNAs in the circbank database and DEmiRNAs from GSE79481. Similarly, the final mRNAs were obtained by selecting overlapping mRNAs in the miRDB database and DEmRNAs from GSE58667. Further investigation was carried out solely on the final miRNA and mRNA targets.

### Establishment of the circRNA–miRNA–mRNA network

We established the circRNA-miRNA-mRNA regulatory network by integrating the potential circRNA-miRNA pairs and miRNA-mRNA pairs. The Cytoscape 3.8.2 software [16] was employed to visualize the ceRNA network.

### Functional enrichment

DAVID is an internet-based biological information database utilized for annotation, visualization, and integrated discovery [17]. KEGG is an integrated database resource for biological interpretation of genome sequences and other high-throughput data. Meanwhile, Gene Ontology (GO) is a fundamental bioinformatics tool utilized to annotate genes and scrutinize their biological processes [18].

### Construction of PPI network and identification of hub genes

Using the online search tool, Search Tool for the Retrieval of Interacting Genes (STRING), a protein-protein interaction (PPI) network of the differentially expressed mRNAs (DEmRNAs) was constructed. The PPI network was then presented visually through the utilization of Cytoscape (version 3.8.2). Furthermore, the identification of hub genes was performed through the use of the Molecular Complex Detection (MCODE) application in Cytoscape.

### Profiling enriched immune cells with CIBERSORT

In order to evaluate alterations in immune cell expression and to obtain the distribution of different immune cell types, we applied the CIBERSORTx algorithm online [19]. To conduct this analysis, we downloaded GSE58667 series matrix files in txt format from the NCBI GEO website. We compared immune cell differences between healthy control subjects and JSpA patients.

### CMap analysis

To explore potential therapeutic agents for JSpA, we employed the CMap tool [20]. Specifically, we utilized the online L1000 platform of the Connectivity Map tool to query the 299 identified DEmRNAs. By submitting a list of 167 upregulated and 132 downregulated hub genes, we obtained connectivity scores, which reflect the proximity of the gene expression profiles on a scale from − 1 to 1. A positive score indicates a promoted effect, while a negative score suggests an inhibited effect.

## Results

### Identification of DEcircRNAs, DEmiRNAs and DEmRNAs

**Fig. 1:**
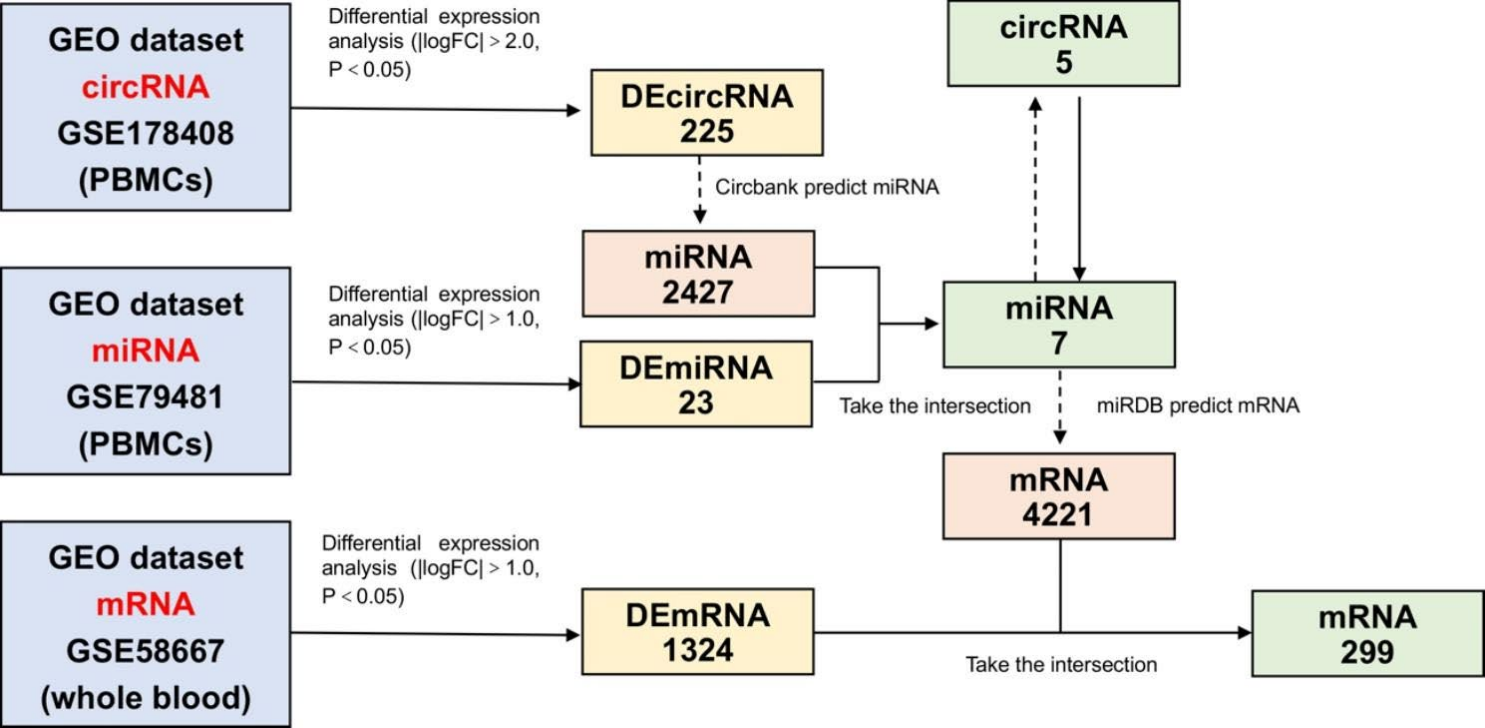
A diagram of workflow

**Fig. 2:**
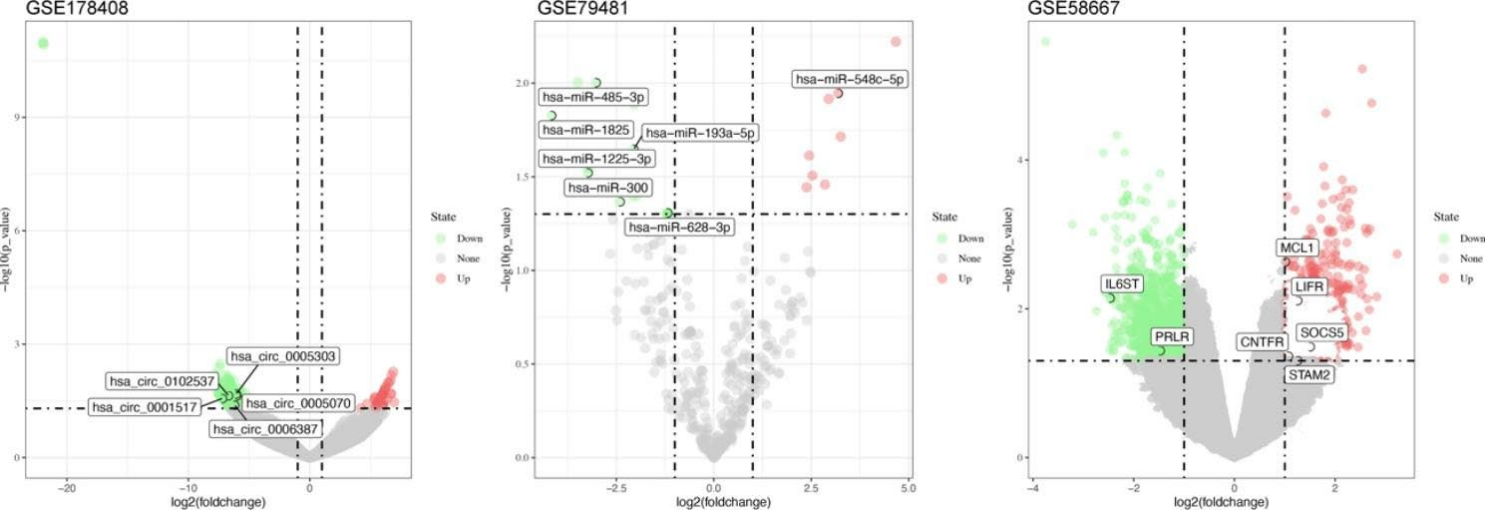
Volcano plots for DEcircRNAs, DEmiRNAs and DEmRNAs.

We performed microarray analysis to determine the expression levels of circRNA, miRNA, and mRNA, as summarized in Table 1. Using a pre-set threshold, we identified 225 DEcircRNAs in the GSE178408 dataset, consisting of 45 upregulated and 180 downregulated circRNAs, 23 DEmiRNAs in the GSE79481 dataset, consisting of 8 upregulated and 15 downregulated miRNAs, and 1324 DEmRNAs in the GSE58667 dataset, consisting of 621 upregulated and 703 downregulated mRNAs. To further analyze the relationship between circRNAs, miRNAs, and mRNAs, we utilized the Circbank and miRDB databases to predict potential interactions. After taking the intersection of the results, we identified 7 DEmiRNAs (1 upregulated and 6 downregulated) and 5 circRNAs that were co-expressed. Finally, we identified 299 co-expressed mRNAs (167 upregulated and 132 downregulated) through the intersection of the 7 miRNAs and 4221 mRNAs obtained from miRDB databases. A flowchart of our analysis is presented in Fig. 1, while the volcano plots of our results are shown in Fig. 2.

**Table 1:** Basic information of the 3 microarray datasets from GEO.

Data source	Platform	Author	Year	Region	Sample size (Disease/Control)	RNA type	Adult/children (Age)	Type of cell
GSE178408	GPL11154	Jun Xiao	2022	China, Guangzhou	6/3	circRNA	Adult (19-44years)	PBMCs
GSE79481	GPL17841	Sushma Singh	2017	India, Uttar Pradesh	8/8	miRNA	Children (13-21years)	PBMCs
GSE58667	GPL570	Fran Borovecki	2014	Croatia, Zagreb	11/4	mRNA	Children (5-17years)	Whole blood

Note: circRNA: Circular RNA, PBMCs: peripheral blood mononuclear cells

### Establishment of the circRNA–miRNA–mRNA network

**Fig. 3:**
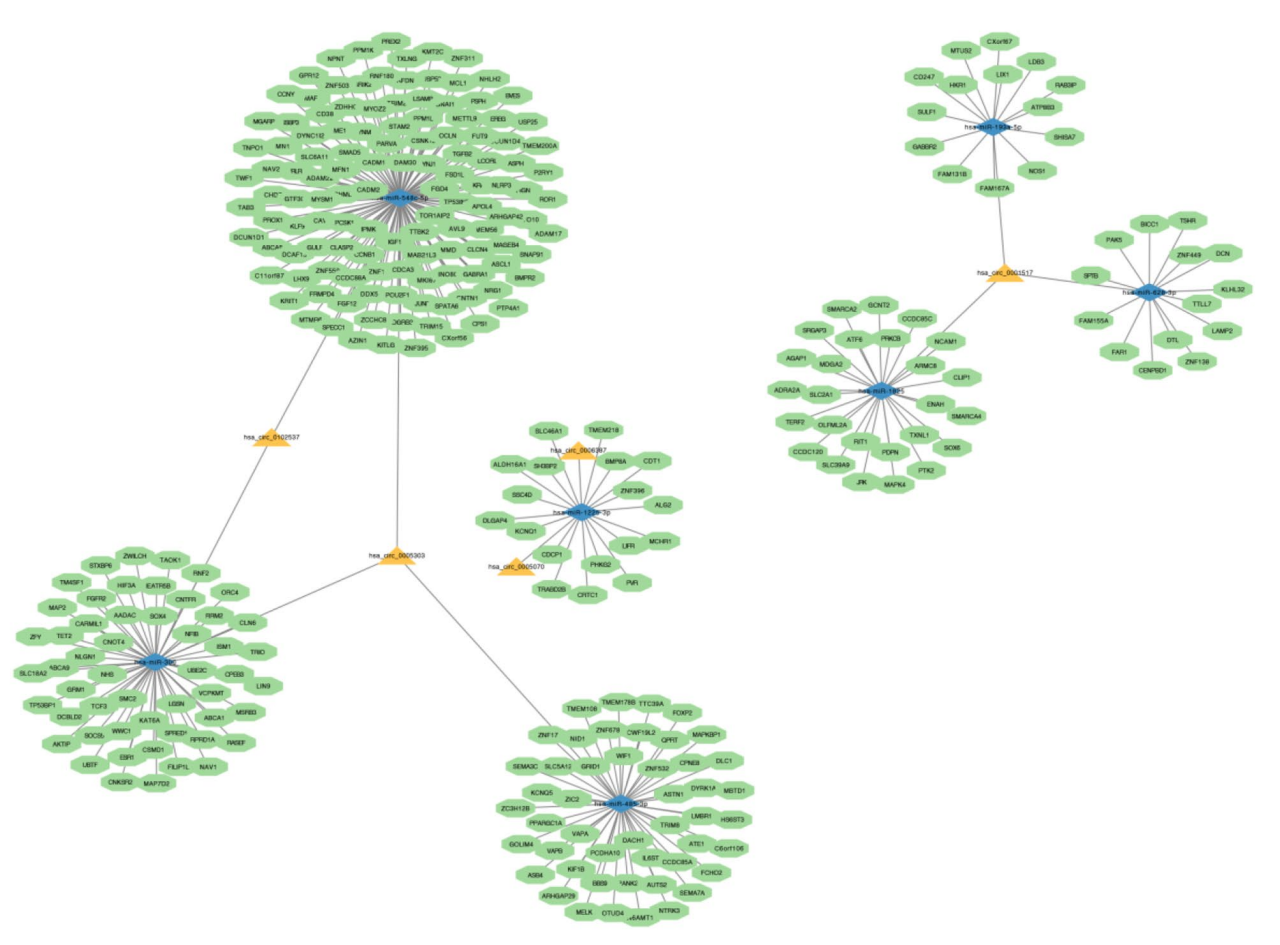
A circRNA–miRNA–mRNA network in JSpA.

In our analysis, we identified 10 interactions between circRNA and miRNA, which consisted of 5 circRNAs (hsa_circ_0006387, hsa_circ_0005070, hsa_circ_0001517, hsa_circ_0005303, and hsa_circ_0102537) and 7 miRNAs (hsa-miR-1225-3p, hsa-miR-1825, hsa-miR-193a-5p, hsa-miR-300, hsa-miR-485-3p, hsa-miR-548c-5p, and hsa-miR-628-3p). We merged the predicted mRNAs of these 7 miRNAs from miRDB with the DEmRNAs retrieved from the GEO database, resulting in a total of 299 target mRNAs. Finally, we integrated the 5 circRNAs, 7 miRNAs, and 299 target mRNAs into a circRNA-miRNA-mRNA network for further investigation, as depicted in Fig. 3. The 5 overlapped circRNAs are summarized in Table 2.

**Table 2:** Basic information on the 5 DEcircRNAs.

CircRNA ID	Position	Genomic length	Strand		Best transcript	Gene symbol	Regulation
hsa_circ_0006387	chr4:89570990–89,579,642	920	+	NM_014606		HERC3	DOWN
hsa_circ_0005070	chr8:103341317–103,357,773	590	-	NM_015902		UBR5	DOWN
hsa_circ_0001517	chr5:107677627–107,684,231	6604	-	NM_001163315		FBXL17	DOWN
hsa_circ_0005303	chr1:41651792–41,660,071	8279	-	NM_001172218		SCMH1	DOWN
hsa_circ_0102537	chr14:71880664–71,884,913	4249	+	None		None	DOWN

### GO term and KEGG pathways enrichment analysis

**Fig. 4:**
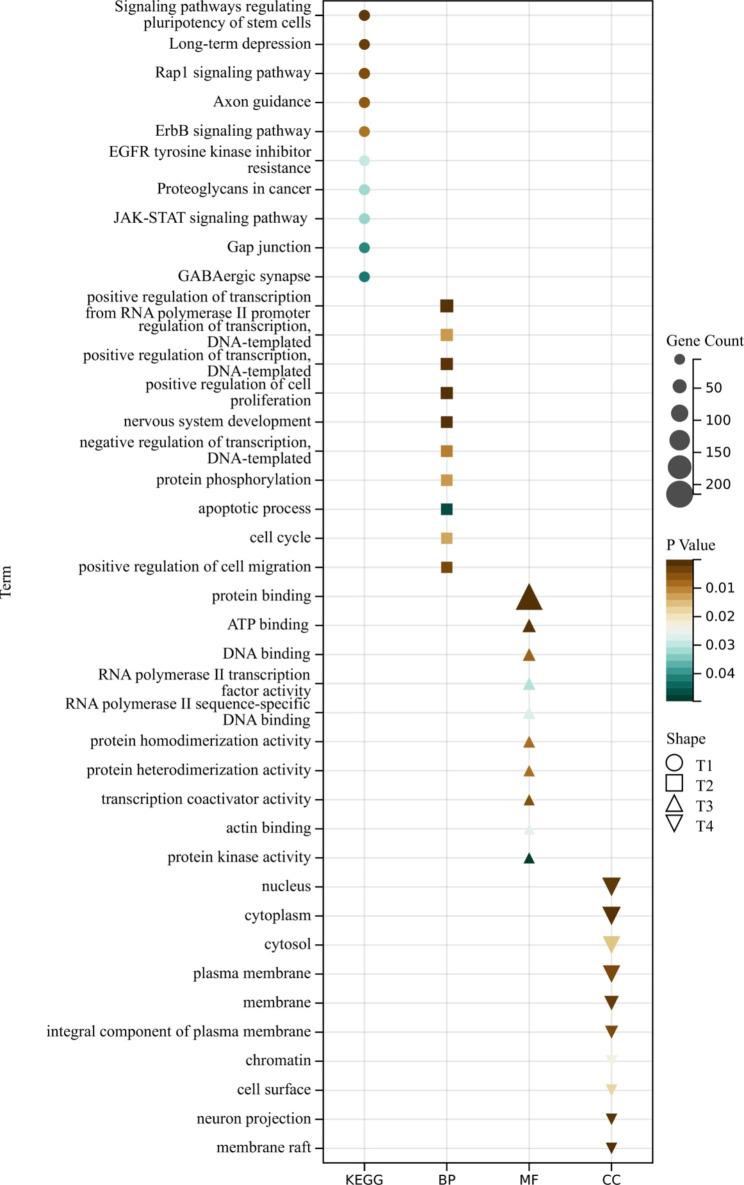
GO annotation and KEGG pathway analyses

Functional and pathway enrichment analyses were conducted using DAVID to obtain a better understanding of the biological roles of the 299 DEmRNAs. Figure 4 displays the enriched KEGG pathways and GO terms. KEGG pathway analysis revealed that the DEGs were mainly associated with the JAK-STAT signaling pathway. Additionally, GO biological process analysis indicated that these DEmRNAs were significantly involved in the regulation of transcription from RNA polymerase II promoter, regulation of transcription and DNA-templated, signal transduction, and positive regulation of cell proliferation. Changes in cellular component were mainly related to the nucleus, cytosol, and cytoplasm. Changes in molecular function were primarily associated with protein binding.

### Establishment of PPI network and identification of hub genes

**Fig. 5:**
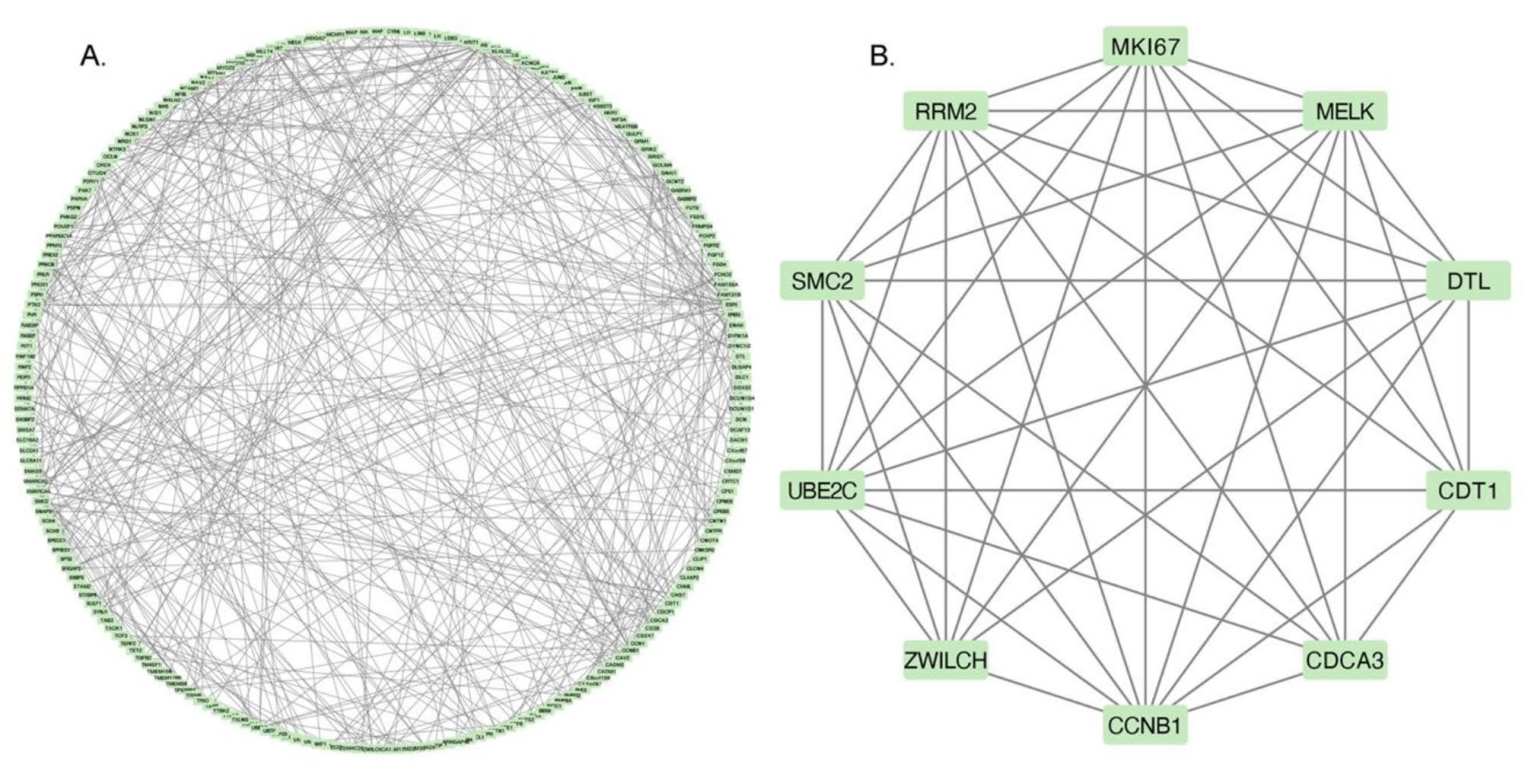
PPI network and the significant module of DEmRNAs. (**A**) The PPI network of DEmRNAs. (**B**) The hub genes were analysed by Cytoscape MCODE

The PPI network analysis of the 299 DEmRNAs resulted in a complex network consisting of 299 nodes and 307 edges, as depicted in Fig. 5A. To identify the most crucial nodes in the network, we employed Cytoscape MCODE, which revealed 10 hub genes. The partial genes are shown in Fig. 5B.

### Enriched immune cells in the pathogenesis of JSpA

**Fig. 6:**
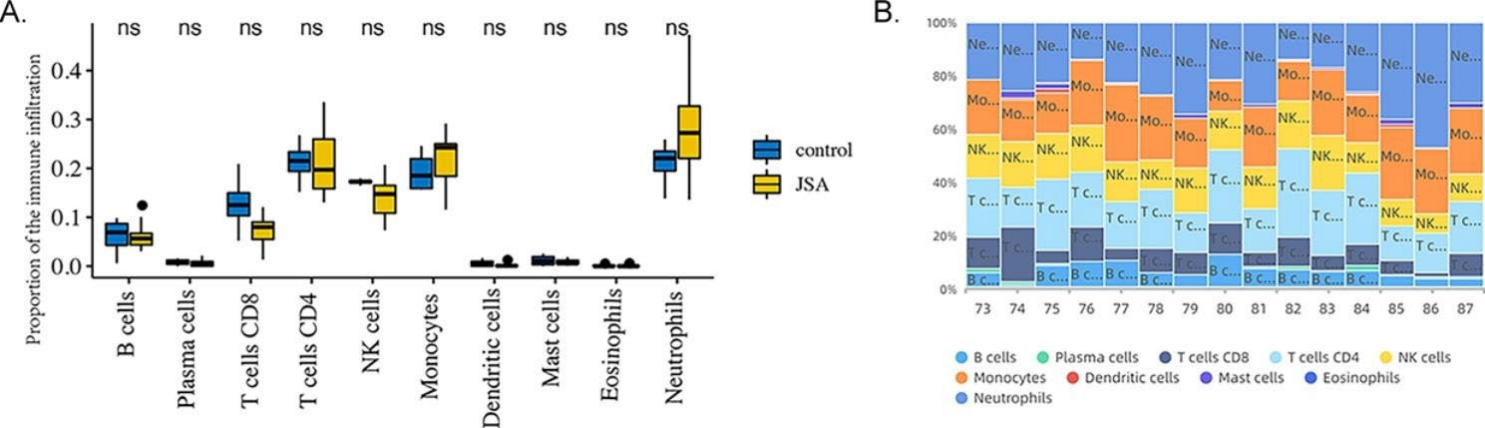
Enriched immune cells in the pathogenesis of JSpA (**A**) The differences of immune cells between JSpA and normal groups. (**B**) Stacked bar charts of immune cell proportions

We can conclude that neutrophils accounted for the majority of all enriched cells, followed by monocytes, NK cells and T cells. Low percentage of B cell and plasma cell infiltration. While, no differential expression proportion of immune- enriched cells in the JSpA and normal groups is shown in Fig. 6.

### Identification of bioactive compounds by CMap analysis

It is important to note that while these compounds (Table 3) may show potential in targeting the DEmRNAs associated with JSpA, further research is needed to determine their safety and efficacy in treating the disease. In addition, it is necessary to conduct extensive pre-clinical and clinical studies before these compounds can be used as a treatment option for JSpA patients.

**Table 3:** Top 10 compounds predicted to have activity against JSpA as predicted via connectivity map

ID	Cmap name	Dose	Cell	Score	Description	Target
BRD-K73794685	azithromycin	0.125 μm	THP1	-0.45	Bacterial 50 S ribosomal subunit inhibitor	MLNR
BRD-K59037100	oxybenzone	0.125 μm	THP1	-0.44	Lipase inhibitor	LIPE
BRD-A68009927	daunorubicin	0.08 μm	THP1	-0.43	RNA synthesis inhibitor |Topoisomerase inhibitor	TOP2A|TOP2B
BRD-K76205745	losartan	0.25 μm	THP1	-0.43	Angiotensin receptor antagonist	AGTR1
BRD-K93208532	apremilast	2.22 μm	THP1	-0.43	Phosphodiesterase inhibitor	PDE4A|PDE4B|PDE4C|PDE4D|TNF|TNFRSF1A
BRD-K49055432	A-66	0.04 μm	THP1	-0.42	PI3K inhibitor	PIK3CA
BRD-K64990520	apocynin	0.74 μm	THP1	-0.42	NADPH inhibitor	NOX3|NOX4
BRD-K89732114	trifluoperazine	10 μm	THP1	-0.41	Dopamine receptor antagonist	DRD2|ABCG2|CAMK2A|SCN4A|SCN9A|ADRA1A|CALM1|CALY|DRD4|HRH1|HTR2A|HTR2C|S100A4|TNNC1
BRD-A49399758	desmopressin-acetate	0.37 μm	THP1	-0.41	Vasopressin receptor agonist	AVPR2|AVPR1A|AVPR1B|OXTR
BRD-K59715032	pasireotide	10 μm	THP1	-0.4	Somatostatin receptor agonist	SSTR1|SSTR2|SSTR3|SSTR5|GH1

## Discussion

The expression patterns of circRNAs have become increasingly noteworthy in various diseases [21, 22]. A significant proportion of circRNAs are concentrated in peripheral blood, peripheral blood mononuclear cells, platelets, plasma, spinal ligament tissues, and bone marrow-derived mesenchymal stem cells, indicating potential functions in spondyloarthritis. Broadly speaking, the dysregulation of circRNAs may have an impact on fundamental molecular mechanisms that are involved in the development of ankylosing spondylitis (AS) [23]. Therefore, a more profound comprehension of the role of circRNAs in JSpA is required.

This study aimed to investigate the impact of circRNAs on the pathogenesis and treatment of JSpA. By conducting a microarray data analysis, we established a circRNA-miRNA-mRNA network and identified mRNAs that were significantly altered, enabling us to identify relevant GO terms and the KEGG pathway. Subsequently, we identified hub genes through the use of the PPI network. Additionally, we discovered several effective compounds, which could offer a novel approach to treating JSpA.

Based on the results of our functional enrichment analysis, we propose that in JSpA, the aberrantly expressed circRNAs utilize both protein and RNA binding to affect the regulation of transcription from the RNA polymerase II promoter, transcription and DNA-templated processes, signal transduction, and cell proliferation. Our KEGG pathway analysis indicates that the differentially expressed genes (DEGs) are primarily associated with the JAK-STAT signaling pathway. The JAK/STAT signaling pathway is a widely expressed intracellular signal transduction pathway that plays a critical role in numerous biological processes, such as cell proliferation, differentiation, apoptosis, and immune regulation. It provides a direct mechanism for extracellular factors to regulate gene expression. Recent studies have focused on the inflammatory effects of this pathway [24], as the JAK-STAT pathways mediate signaling for multiple cytokines, including those implicated in the pathogenesis of spondyloarthritis (SpA) [25].

Previous studies have shown the significance of circRNAs in the pathogenesis of AS. For example, Wang T et al. identified two down-regulated circRNAs (circPTPN22 and circFCHSD2) and constructed a circRNA-miRNA-mRNA regulatory network based on these two circRNAs [5]. Additionally, Kou J found that differentially expressed circRNAs play crucial roles in AS and constructed circRNA-miRNA regulatory networks [6]. Other studies have also demonstrated the involvement of circRNAs in the development of AS. For instance, Song M et al. discovered that hsa_circ_0000652 aggravates inflammation by activating macrophages and enhancing OX40/OX40L interaction in AS [7]. Tang YP found that hsa_circRNA_012732 has the potential to be an indicator of disease activity, while hsa_circRNA_001544 could serve as a molecular marker for AS diagnosis [8]. Furthermore, Wang S identified hsa_circ_0070562 as a pro-osteogenic factor in AS [9].

In our study, we identified five circRNAs (hsa_circ_0006387, hsa_circ_0005070, hsa_circ_0001517, hsa_circ_0005303, hsa_circ_0102537) in the final circRNA-miRNA-mRNA network. To our knowledge, there have been no relevant studies on the potential functions of the top four circRNAs in JSpA or other diseases. However, a study on lung adenocarcinoma (LUAD) identified hsa_circ_0102537 to be downregulated in LUAD plasma exosomes and tissues, suggesting that it could be involved in LUAD progression [26]. Regarding miRNAs, the seven miRNAs identified in our study have been widely reported in various diseases [27–31]. For example, hsa-miR-1825 was found to be down-regulated in patients with common variable immunodeficiency (CVID) who received Ig infusion [32], while plasma miR-628-3p was upregulated in atopic dermatitis (AD) patients with severe atopic keratoconjunctivitis (AKC) [33]. However, except for the above two miRNAs, there have been no relevant studies on the remaining miRNAs in JSpA or other rheumatic immune diseases.

Through CMap analysis, we identified ten drugs (azithromycin, oxybenzone, daunorubicin, losartan, apremilast, A-66, apocynin, trifluoperazine, desmopressin-acetate, and pasireotide) as potential treatment options for JSpA. Previous studies have shown that the PI3K/AKT pathway plays a significant role in the pathogenesis of AS, and the PI3K pathway is highly active in this disease [34–36]. A-66 is a PI3K p110α isoform-selective inhibitor that has been found to inhibit cell growth in melanomas [37]. Although there are no reports on the effectiveness of A-66 in the treatment of JSpA, we hypothesize that it could be a potential therapeutic option for this disease.

The construction of a circRNA-miRNA-mRNA network in this study has illuminated the regulatory mechanisms of juvenile spondyloarthropathies (JSpA) and has yielded valuable insights into the underlying molecular mechanisms and potential therapeutic targets for this disease. Nevertheless, it is important to acknowledge the limitations inherent in this study. Due to the absence of our own sequencing data, this study relied on existing datasets for analysis, which may have introduced certain limitations and constraints. For example, patients from GSE178408 were not JspA, and three datasets were obtained from different biological tissues or sources. However, this study serves as a springboard for future exploration. It has highlighted the need for subsequent experiments to be conducted in future studies, where independent sequencing data can be generated to further validate and expand upon the findings presented here.

## Conclusions

To summarize, the results of our study provide valuable information regarding the regulatory mechanisms of JSpA through the establishment of a circRNA-miRNA-mRNA network using microarray data and comprehensive bioinformatics analyses. This study sheds light on potential therapeutic targets for JSpA, and may facilitate the development of new treatment strategies.

## Electronic supplementary material

Below is the link to the electronic supplementary material.

Supplementary Material 1

Supplementary Material 2

## Data Availability

The data and materials can be obtained in https://www.ncbi.nlm.nih.gov/geo/.

## List of abbreviations

JSpA: Juvenile spondyloarthropathies
GEO: Gene Expression Omnibus
PPI: Protein-protein interaction
DEcircRNAs: Differentially expressed circRNAs
DEmiRNAs: Differentially expressed miRNAs
DEmRNAs: Differentially expressed mRNAs
ceRNA: Competitive endogenous RNA
circRNA: Circular RNA
AS: Ankylosing spondylitis
PBMCs: Peripheral blood mononuclear cells
CMap: Connectivity map
GO: Gene Ontology
STRING: Search Tool for the Retrieval of Interacting Genes
MCODE: Molecular Complex Detection
DEGs: Differentially expressed genes
SpA: Spondyloarthritis
LUAD: Lung adenocarcinoma
CVID: Common variable immunodeficiency
AD: Atopic dermatitis
AKC: Atopic keratoconjunctivitis
