# Supplementary figures and images for: Identifying circRNA-associated-ceRNA networks in juvenile spondyloarthropathies patients
Source: Pediatr Rheumatol Online J. 2023 Jul 28;21:75. doi: 10.1186/s12969-023-00855-2 (PMC10386608; doi:10.1186/s12969-023-00855-2)

GSE178408

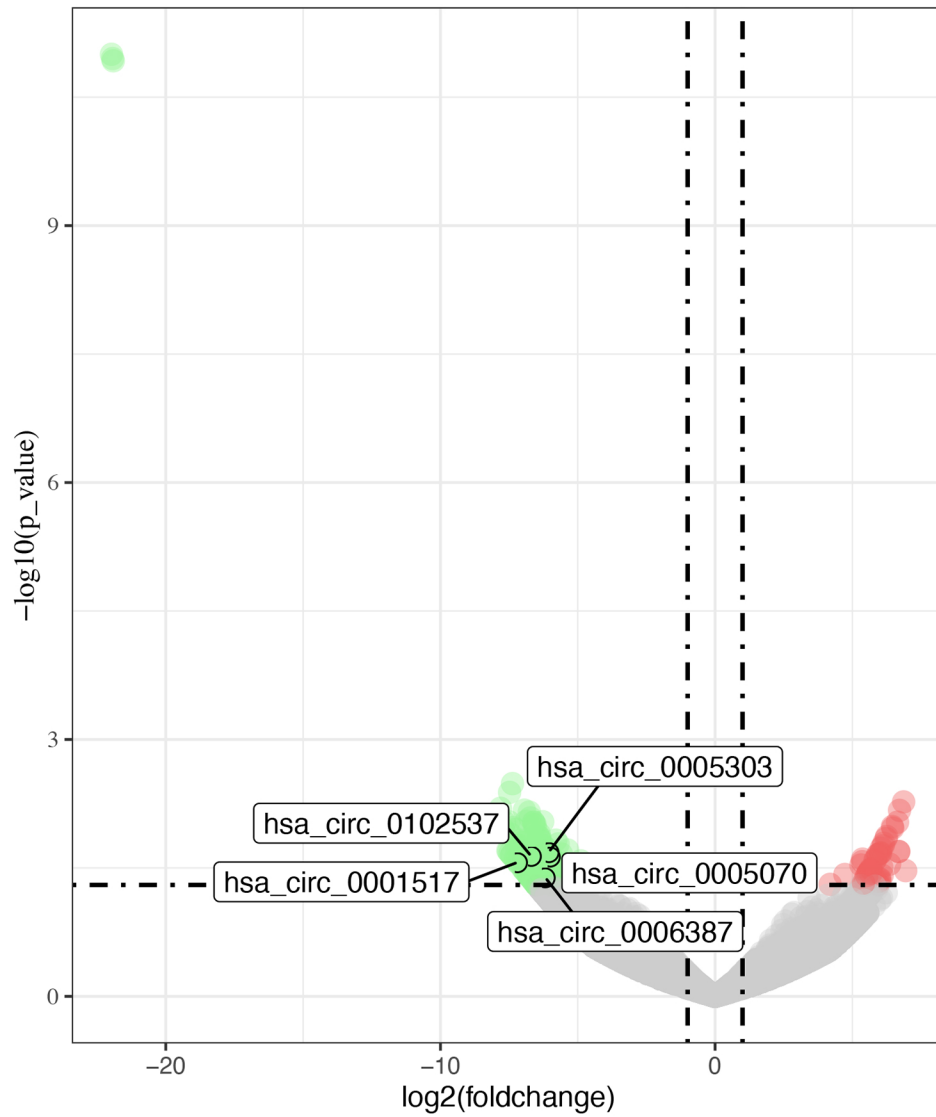

GSE79481

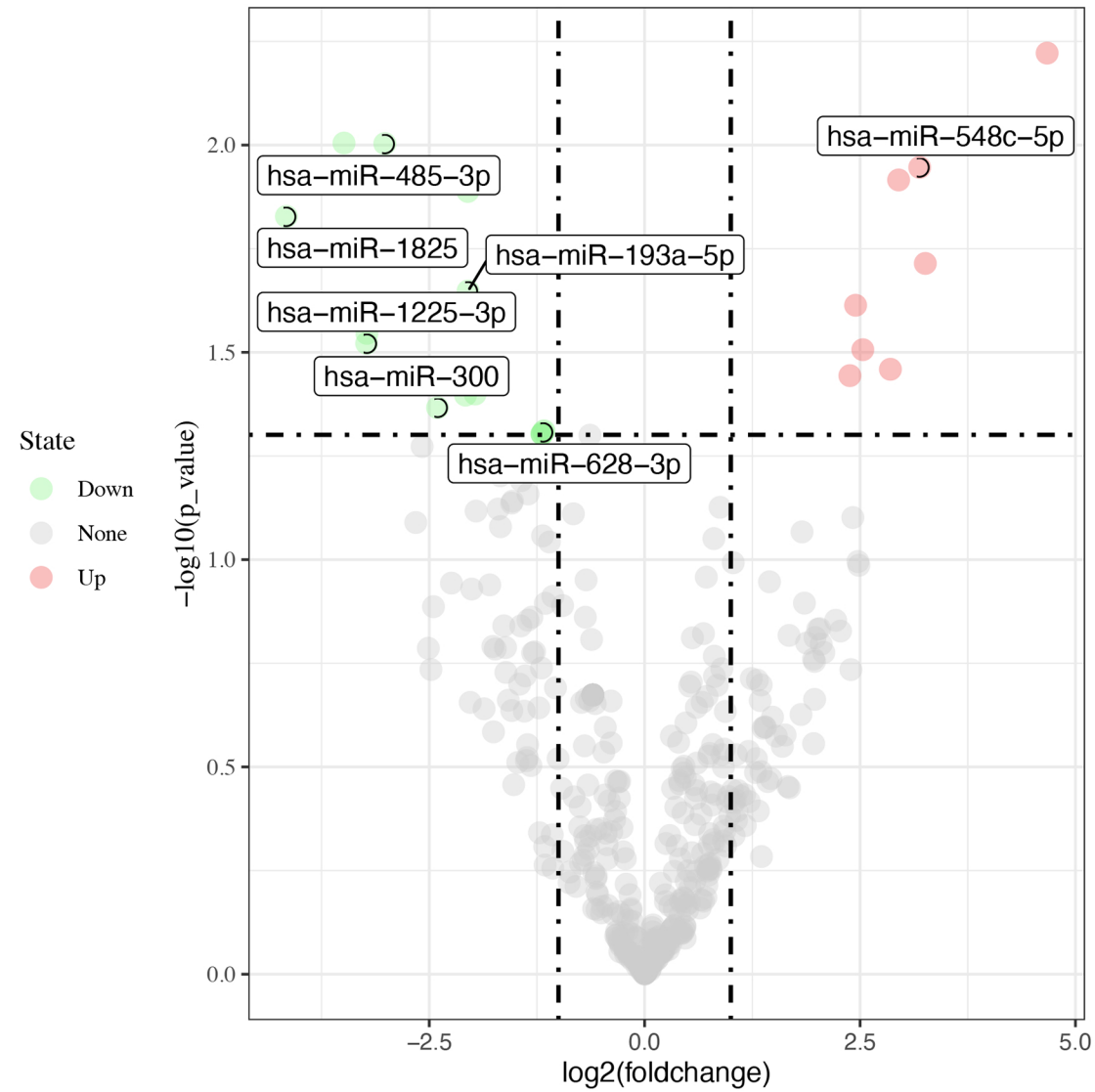

GSE58667

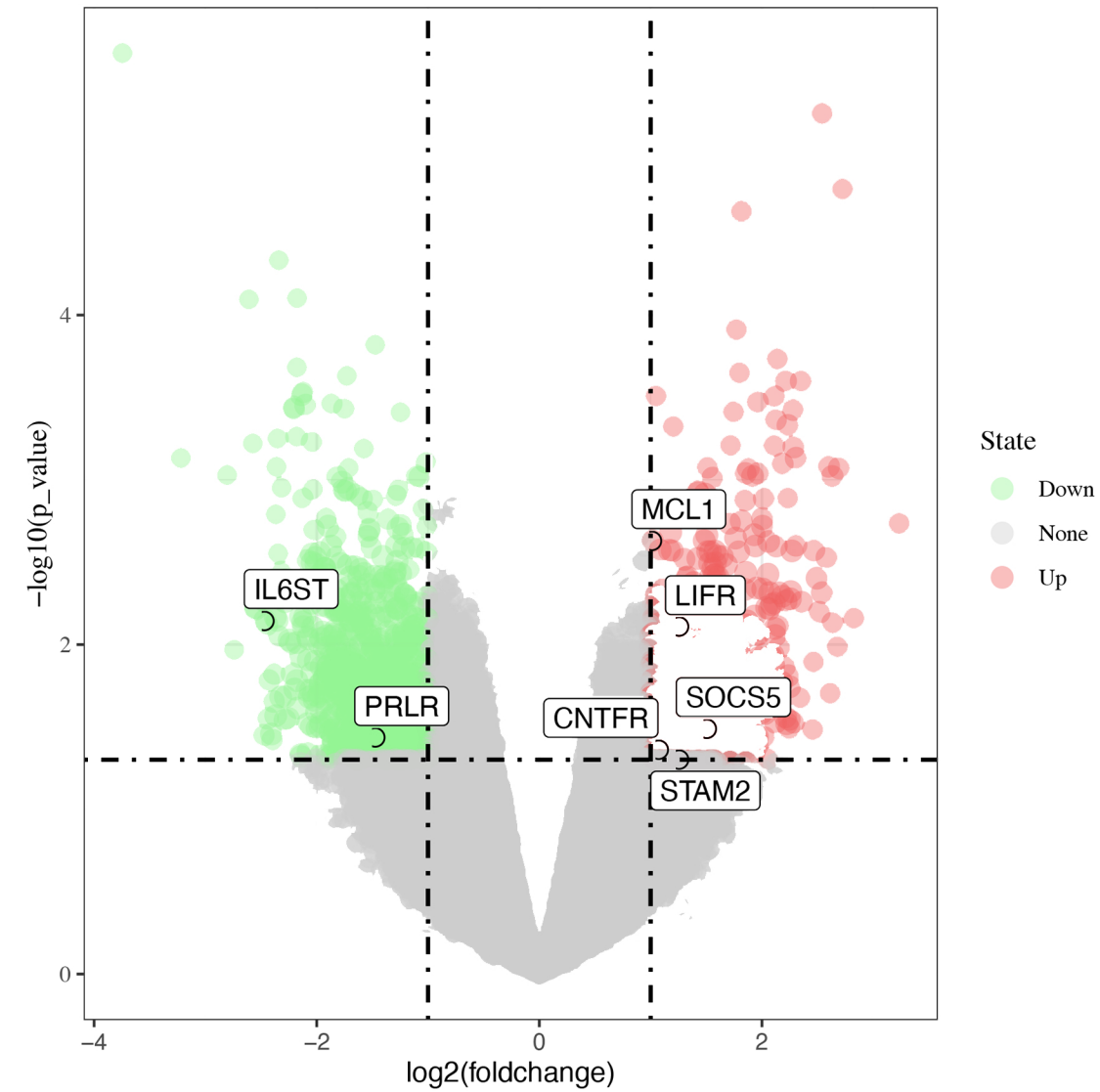

Supplement: Supplementary file 1 — Supplementary Material 1 [file 12969_2023_855_MOESM1_ESM.pdf]

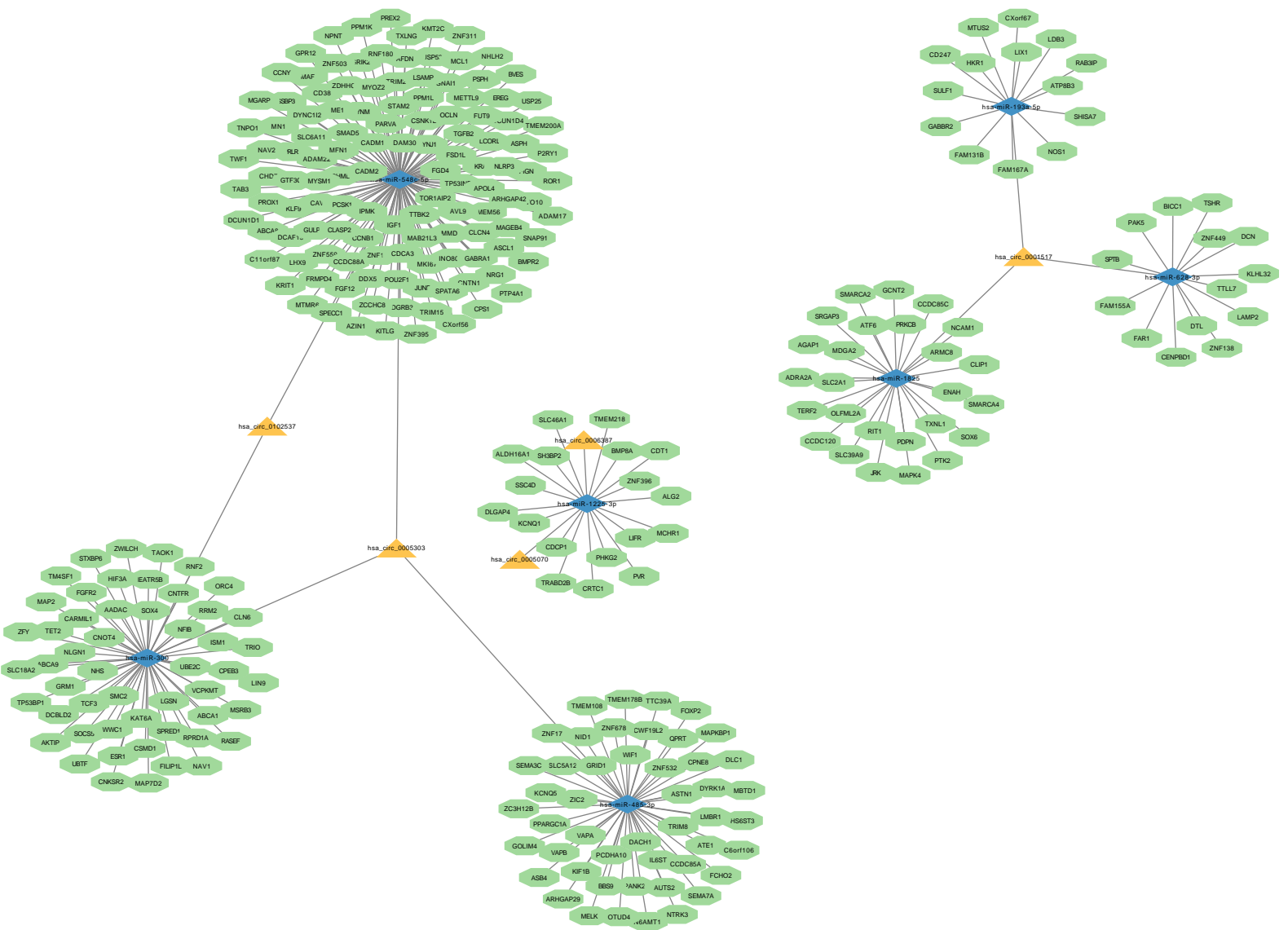

Supplement: Supplementary file 2 — Supplementary Material 2 [file 12969_2023_855_MOESM2_ESM.pdf]
